# Comparison of Centella with Flavonoids for Treatment of Symptoms in Hemorrhoidal Disease and After Surgical Intervention: A Randomized Clinical Trial

**DOI:** 10.1038/s41598-020-64772-0

**Published:** 2020-05-14

**Authors:** Massimo Chiaretti, Danilo Alunni Fegatelli, Giuseppe Pappalardo, Michele Dello Spedale Venti, Annalisa Italia Chiaretti

**Affiliations:** 1 Department of General Surgery and Specialties “Paride Stefanini”, Sapienza University of Rome, Rome, 00161 Italy; 2 Department of Public Health and Infectious Diseases, Sapienza University of Rome, Rome, 00161 Italy; 3 Department of Molecular Medicine, Sapienza University of Rome, Rome, 00161 Italy

**Keywords:** Anal diseases, Anal diseases, Anus, Randomized controlled trials

## Abstract

Phlebotonics’ effects were evaluated to reduce time-to-stop bleeding and anal irritation in 130 patients who complained of hemorrhoidal disease (HD); bleeding and pain after hemorrhoidectomy (31 patients) and hemorrhoidal thrombosis (34 patients) in the short time. Sixty patients were randomized to receive the routine treatment (both conservative and surgical) (control Group C). The treated group (both conservative and surgical) was divided into two subgroups: one treated with flavonoids (Group A, n = 73), the other with Centella (Group B, n = 66). Time-to-stop bleeding was checked at baseline and checkups (0 up to day 42). Healing was estimated with Kaplan-Meier method, the Kruskal-Wallis test estimated changes in the VAS scores. The HD median time-to-stop bleeding was 2 weeks for Groups A and B; 3 weeks for Group C. VAS scores comparison among Groups (irritation): A vs C, p = 0.007; B vs C, p = 0.041; and A vs B, p = 0.782 resulted respectively. As for operated hemorrhoids, the time-to-stop bleeding was 3 and 4 weeks in Groups A and B and 5 in Group C. Histopathology showed an association between flavonoids and piles’ fibrosis (p = 0.008). Phlebotonics in HD, as well as after surgery, showed significant beneficial effects. Flavonoids are the most effective phlebotonics against bleeding and anal irritation.

## Introduction

The hemorrhoidal disease (HD) has a general population prevalence ranging from 13% to 36%^1^ with an estimated incidence of approximately 50% between 45 and 65 aged^2^. HD appears with symptoms and signs of soiling, itching, pain, prolapse, and defecation bleeding that are commonly associated with enlarged hemorrhoidal cushions. It may also be symptomatic of other diseases^3,4^. Anal irritation in the anorectal region can be due to fissure, anal itching, diabetes skin tags, yeast infection, acquired immunodeficiency disease syndrome, herpetic infection^5^, allergic or irritant dermatitis, and fungal infections on the anus skin^6,7^.

Hemorrhoids are anal vascular cushions needed to ensure complete closure of the anus to gas, and liquid stools^4^. Varicose dilatation of the hemorrhoids often develops from a persistently elevated venous pressure within the vascular plexus^1–4,6^. Etiology seems to relate to triggering factors (i.e. constipation that affects many women but not all women) and the predisposition grounds (i.e. menstrual period). In fact, many women develop piles during menstrual period and pregnancy^6–8^ (up to 80%). The hormones and the oral contraceptive pill’s intake seem to facilitate HD and acute hemorrhoidal crisis^3,4,8^. Moreover, age, poverty related factors, and low-in-water and low-in-vegetable-fibers diets promote constipation^9–12^, that is related to the start of HD^1–4^. Sedentary jobs can cause a difficult plexus discharge, a pressure increasing, and dilatation of cushions. The conservative management (diet rich in water and fibers, stool softeners and hygienic cares) is a possible HD treatment from I to III grade in Golligher’s classification^1–4,9–11^. Furthermore, there are a lot of therapies available for bearings’ treatment, both surgical and conservative^13–21^.

Among conservative available therapies, we evaluated the effects of two phlebotonics (flavonoids and Centella)^13–22^ in comparison between them and with the control group.

The involvement of free radicals in the hemorrhoids’ precipitation is *“a consequence of improper balance between reactive oxygen species and their metabolites*^23^*”*. It is known that free radicals are neutralized by antioxidants contained in phlebotonics. This is a therapeutic effect on the HD management.

As stated by Evans and Halliwell, “*herbal extracts rich in phytoantioxidants like polyphenols, flavonoids and other related compounds are known to possess positive health effects and eventually reduces the incidence of diseases*^24^*”*. Therefore, many researchers focused their attention “*on the use of natural antioxidants, that can provide more significant health benefits with minimal toxicities*^25–27^*”* as observed also in our own experience.

Perera *et al*. defined phlebotonics as “*a heterogeneous class of drugs consisting of plant extracts (i.e. flavonoids*^13–20^, Centella asiatica^21,22^) *and synthetic compounds (i.e. calcium dobesilate*)^14^”. Corsale *et al*. noticed that phlebotonics in HD therapy *“improve venous tone, stabilize capillary permeability and increase the lymphatic”* and the hemorrhoidal “*drainage*^13–15^*”*.

Anti-inflammatory activities of flavonoids are widely studied. Faujdar *et Al*. reported that “*oxaprozin binding mode is compared to the active site of cycloxygenase enzyme (COX) which is responsible for inflammatory mediator’s catalysis*^27^*”*. Moreover, they stated that “*pharmacophore’s structure shows specific features required to bind the inhibitor to the active site of COX*^27^*”*. These researches mapped the oxaprozin structures and noticed that they have the same structure of the well known COX-inhibitor^27^.

Rabiskova *et al*. found out that the *“inhibition of some key enzymes involved in inflammation response explains the anti-inflammatory effect of rutin*^28^”. It promotes colonic healing in inflammatory bowel disease (IBD) by myeloperoxidase activity suppression at a dose of 10 mg/kg^28^. Rutin is a promising preparation, without side effects, suitable for lifelong IBD therapy^28,29^.

In another study, it was proved that rutin inhibits the edema of the ear caused by xilol. Moreover, rutin reduces cell migration in both peritonitis (carrageenan-induced) and air pouch (zymosan-induced) based on animal models. The study also showed reduced levels of cytokines^30^.

Yoo, Ku, Baek, and Bae found that rutin potently inhibits HMGB1 release. In particular, the rutin reduces HMGB1 inflammatory response in human endothelial cells. Similarly, rutin reduces vascular hyper permeability and leukocyte migration caused by HMGB1in mice model^31^.

Kim *et al*. demonstrated that *“rutin has an anti-inflammatory effect and it might protect against allergic rhinitis*^32^*”*. For the first time, they showed that *“rutin suppresses chemokines (ICAM-1 and MIP-2) and the reduction of inflammatory cells by regulating the levels of the vascular endothelial growth factor (VEGF)*^”32^. In addition, they observed that *“rutin reduces inflammatory cytokines and the activation of caspase-1*^32^*”*.

“Hydroxyethylrutosides (HR), also known as oxerutins, is a mixture of semi-synthetic flavonoids obtained through the hydroxylation of rutin^33^”. Antignani et al. demonstrated that “the most important pharmacologic action of oxerutins is the inhibitory effect on microvascular permeability^33^”. This effect allows to reduce the development of edema that follows several types of injury^33^. Petruzzellis et al. demonstrated “the edema’s reduction in controlled double blind clinical trials, both in healthy volunteers and in patients with chronic venous insufficiency (CVI)^34^”. They concluded that “considering both noninvasive tests and clinical evaluation, oxerutin is effective in controlling chronic venous hypertension, without side effect, and with good tolerability^34^”.

Aziz *et al*. carried out a systematic review of oxerutins efficacy and tolerability referring to signs and symptoms in CVI^35^. Their findings showed that oxerutins produces “*modest improvements in several CVI symptoms*^35^”.

In a review on fourteen trials, Alonso-Coello compared “*the use of phlebotonics versus placebo or without treatment in HD*^8^”. Literature shows that purified micronized flavonoids fraction (PMFF) reduces the severity of bleeding and prevents its relapse^8,13–20,36^. Numerous trials, assessing the effects of phlebotonics compounds in treating HD, suggest that they are able to obtain better results^3,4,8,13–22,36^.

*Cho*^20^ and other researchers^21,22^ noticed that, aside from flavonoids, Centella asiatica (Ca) stimulates collagen type-1 synthesis, production and accumulation of new extracellular matrix. Ca improves vascular-connective tissue deposition and repair processes, which act on enzymatic hydrolysis of microbes and leucocytes to shield collagen^20–22^.

On the basis of these considerations, we thought it could be useful to carry out a randomized trial on the clinical effects of flavonoids^3,4,8,13–20^ and Ca^21,22^ (treated Group) in comparison to a control group in HD patients. The same study was performed on surgical patients (i.e. post-hemorrhoidectomy and thrombosis).

## Results

### Subacute and chronic symptomatology of grades II-III-IV HD patients (n = 130)

The upper section of Table 1 shows features of hundred thirty patients (65.3%) afflicted with II-III-IV Golligher’s grade piles and subacute and chronic symptomatology.

**Table 1:** Baseline characteristics of investigated population.

Population enrolled with HD	Total (n = 199)	Group A (n = 73)	Group B (n = 66)	Group C (n = 60)
Subacute and chronic symptomatology grades II-III-IV HD (n = 130)	Total (n = 130) (65.3%)	Group A (n = 45)	Group B (n = 46)	Group C (n = 39)
Sex (male)	73 (56.2%)	34 (75.6%)	21 (45.7%)	18 (46.2%)
Age (years)	51.0 ± 14.1	49.3 ± 13.0	48.8 ± 14.5	55.6 ± 14.1
Grade II	11 (8.5%)	5 (11.1%)	3 (6.5%)	3 (7.7%)
Grade III	59 (45.4%)	22 (48.9%)	19 (41.3%)	18 (46.2%)
Grade IV	60 (46.2%)	18 (40.0%)	24 (52.2%)	18 (46.2%)
**bleeding**	136 (68.3%)	50 (68.5%)	45 (68.2%)	41 (68.3%)
irritation	184 (92.5%)	68 (93.2%)	62 (93.9%)	54 (90.0%)
**Bleeding grade III-IV hemorrhoids underwent hemorrhoidectomy (n = 31)**	**Total (n = 31)** **(15.6%)**	**Group A (n = 10)**	**Group B (n = 9)**	**Group C (n = 12)**
Sex (male)	27 (87.1%)	7 (70.0%)	8 (88.9%)	12 (100.0%)
Age (years)	49.7 ± 11.5	47.6 ± 11.8	50.6 ± 13.2	50.7 ± 10.5
**bleeding**	26 (83.9%)	8 (80.0%)	6 (66.7%)	12 (100.0%)
pain	30 (96.8%)	9 (90.0%)	9 (100.0%)	12 (100.0%)
**Bleeding grade II hemorrhoid patients underwent rubber band ligation (n = 4)**	**Total (n = 4)** **(2.0%)**	**Group A (n = 1)**	**Group B (n = 2)**	**Group C (n = 1)**
Sex (male)	4 (100.0%)	1 (100.0%)	2 (100.0%)	1 (100.0%)
Age (years)	51.7 ± 11.9	52.0	43.5 ± 4.9	68.0
**bleeding**	4 (100.0%)	1 (100.0%)	2 (100.0%)	1 (100.0%)
pain	4 (100.0%)	1 (100.0%)	2 (100.0%)	1 (100.0%)
**Hemorrhoidal Thrombosis underwent incision and drainage procedure (n = 34)**	**Total (n = 34) (17.1%)**	**Group A (n = 17)**	**Group B (n = 9)**	**Group C (n = 8)**
Sex (male)	19 (55.9%)	9 (52.9%)	4 (44.4%)	6 (75.0%)
Age (years)	46.0 ± 12.5	46.4 ± 10.4	49.4 ± 14.5	41.4 ± 14.6
**bleeding**	15 (44.1%)	7 (41.2%)	4 (44.4%)	4 (50.0%)
pain	31 (91.2%)	17 (100.0%)	7 (77.8%)	8 (87.5%)

### Sixty nine patients (34.7%) with acute hemorrhoidal symptomatology

Thirty one patients with bleeding grade III-IV HD (15.6%) underwent *hemorrhoidectomy*. Four (2.0%) with bleeding grade II piles underwent rubber band ligation (excluded for statistic evaluation). Thirty four (17.1%) with *hemorrhoidal thrombosis* (HT) underwent incision and drainage procedure (lower section of Table 1).

### Phlebotonics clinical effects in comparison to controls

#### Comparison on 130 patients with grades II-III-IV HD

At the beginning, bleeding was complained in 68.5% patients of Group A, in 68.2% in Group B, and 68.3% in Group C (Table 1).

**Figure 1:**
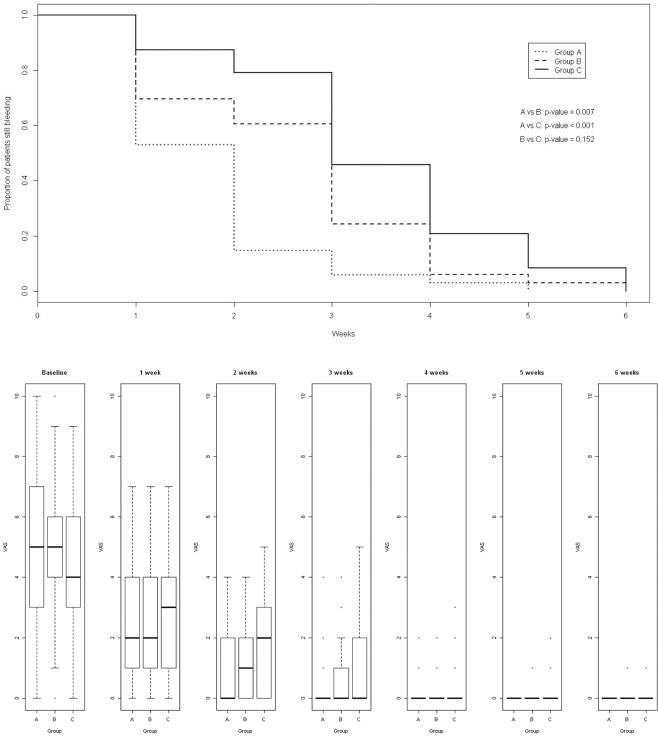
(**A**) (upper) The median time-to-stop bleeding, in 130 patients with Golligher’s hemorrhoids grade II, III and IV was 2 weeks in Group A and 3 weeks in Groups (**B,C**), respectively. (**B**) (lower) VAS scores (anal irritation evaluation) in 130 patients with grade II-III-IV HD at checkpoints.

Median time-to-stop bleeding was 2 weeks in Group A (95% confidence interval, 1–2 weeks), 3 weeks in Groups B and C (95% confidence interval, 2–3 and 3–4 weeks respectively). The log-rank test about bleeding showed some significant differences between Groups (A vs B, p = 0.007; A vs C, p < 0.001; B vs C, p = 0.152) (Fig. 1A).

As for the anal irritation’s scores (VAS), from baseline to the sixth week the Nemenyi test showed differences among Groups A, B, and C (A vs C, p = 0.007; B vs C, p = 0.041; A vs B, p = 0.782) (Fig. 1B and upper section of Table 2).

**Table 2:** VAS scores monitoring of investigated population.

grades II-IV HD (n = 130)	Group A					Group B					Group C				
	Min	Q1	Me	Q3	Max	Min	Q1	Me	Q3	Max	Min	Q1	Me	Q3	Max
Baseline	0.0	3.0	5.0	7.0	10.0	0.0	4.0	5.0	6.0	10.0	0.0	3.0	4.0	6.0	9.0
1 week	0.0	1.0	2.0	4.0	7.0	0.0	1.2	2.0	4.0	7.0	0.0	1.0	3.0	4.0	7.0
2 weeks	0.0	0.0	0.0	2.0	4.0	0.0	0.0	1.0	2.0	4.0	0.0	0.0	2.0	3.0	5.0
3 weeks	0.0	0.0	0.0	0.0	4.0	0.0	0.0	0.0	1.0	4.0	0.0	0.0	0.0	2.0	5.0
4 weeks	0.0	0.0	0.0	0.0	2.0	0.0	0.0	0.0	0.0	2.0	0.0	0.0	0.0	0.0	3.0
5 weeks	0.0	0.0	0.0	0.0	0.0	0.0	0.0	0.0	0.0	1.0	0.0	0.0	0.0	0.0	2.0
6 weeks	0.0	0.0	0.0	0.0	0.0	0.0	0.0	0.0	0.0	1.0	0.0	0.0	0.0	0.0	1.0
p-Value	A vs C, *p* = 0.007 A vs B, *p* = ns					B vs C, *p* = 0.041									
**Hemorrhoidectomy (n = 31)**															
Baseline	3.0	3.0	5.0	7.0	8.0	4.0	6.0	7.0	7.0	8.0	0.0	5.0	5.0	6.2	7.0
1 week	0.0	0.5	2.0	5.5	8.0	2.0	4.0	6.0	8.0	8.0	1.0	2.0	3.50	5.0	7.0
2 weeks	0.0	0.0	0.5	2.0	4.0	1.0	2.0	2.0	5.0	7.0	0.0	0.0	1.0	2.2	7.0
3 weeks	0.0	0.0	0.0	0.0	3.0	0.0	0.0	0.0	1.0	5.0	0.0	0.0	0.0	1.2	4.0
4 weeks	0.0	0.0	0.0	0.0	3.0	0.0	0.0	0.0	1.0	4.0	0.0	0.0	0.0	1.2	3.0
5 weeks	0.0	0.0	0.0	0.0	1.0	0.0	0.0	0.0	0.0	3.0	0.0	0.0	0.0	0.0	2.0
6 weeks	0.0	0.0	0.0	0.0	0.0	0.0	0.0	0.0	0.0	1.0	0.0	0.0	0.0	0.0	2.0
p-Value	A vs C, *p* = 0.045 A vs B, *p* = ns					B vs C, *p* = ns									
**Hemorrhoidal Thrombosis (n = 34)**															
Baseline	4.0	6.0	8.0	9.0	10.0	3.0	4.0	7.0	8.0	10.0	0.0	3.7	5.0	6.7	9.0
1 week	0.0	1.0	2.0	3.0	6.0	0.0	1.0	2.0	3.0	6.0	0.0	1.7	3.0	4.2	9.0
2 weeks	0.0	0.0	0.0	0.0	3.0	0.0	0.0	0.0	1.0	3.0	0.0	0.0	1.0	1.2	5.0
3 weeks	0.0	0.0	0.0	0.0	2.0	0.0	0.0	0.0	0.0	0.0	0.0	0.0	0.0	0.2	2.0
4 weeks	0.0	0.0	0.0	0.0	1.0	0.0	0.0	0.0	0.0	0.0	0.0	0.0	0.0	0.0	1.0
5 weeks	0.0	0.0	0.0	0.0	0.0	0.0	0.0	0.0	0.0	1.0	0.0	0.0	0.0	0.0	0.0
6 weeks	0.0	0.0	0.0	0.0	0.0	0.0	0.0	0.0	0.0	0.0	0.0	0.0	0.0	0.0	0.0
p-Value	A vs C, *p* < 0.001 A vs B, *p* < 0.001					B vs C, *p* < 0.001									

Min = minimum value; Q1 = lower quartile; Me = Median; Q3 = upper quartile; Max = maximum value.

#### Comparison on 31 patients with bleeding grade III-IV HD (underwent hemorrhoidectomy)

**Figure 2:**
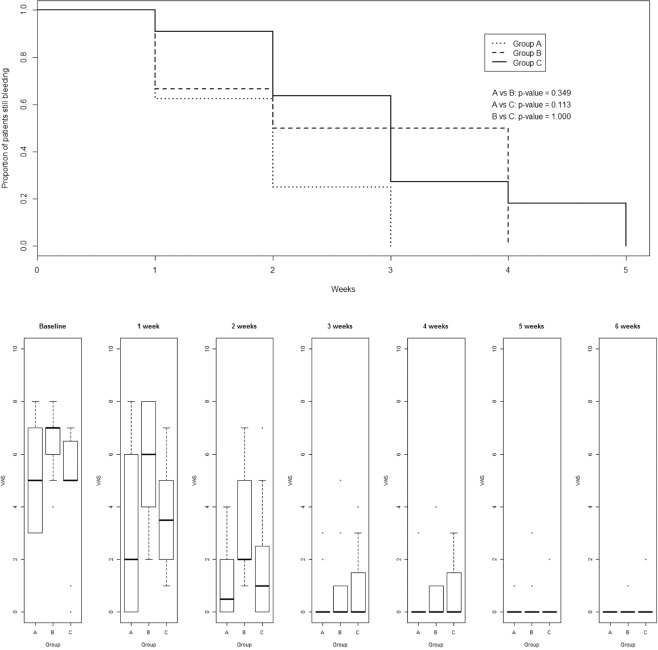
(**A**) (upper) The median time-to-stop bleeding, in 31 patients with grade III-IV bleeding hemorrhoids underwent hemorrhoidectomy was 1 week for Group (**A**) and 2 weeks for both Groups (**B,C**), respectively. (**B**) (lower) VAS scores (pain evaluation) in 31 patients underwent hemorrhoidectomy.

Median time-to-stop bleeding resulted 3 and 4 weeks in Groups A and B respectively; 5 in Group C (Fig. 2A and middle section of Table 1). All patients stopped bleeding within the fifth week, without significant differences among groups (A vs B, p = 0.349; A vs C, p = 0.113; B vs C, p = 1.000). Groups A and B had better trends than Group C.

As for pain in operated patients, statistical difference was detected in VAS scores among Groups A and C (p = 0.045) (Fig. 2B and middle section of Table 2).

#### Comparison on 34 patients with hemorrhoidal thrombosis (HT) (incised and drained in local anesthesia)

**Figure 3:**
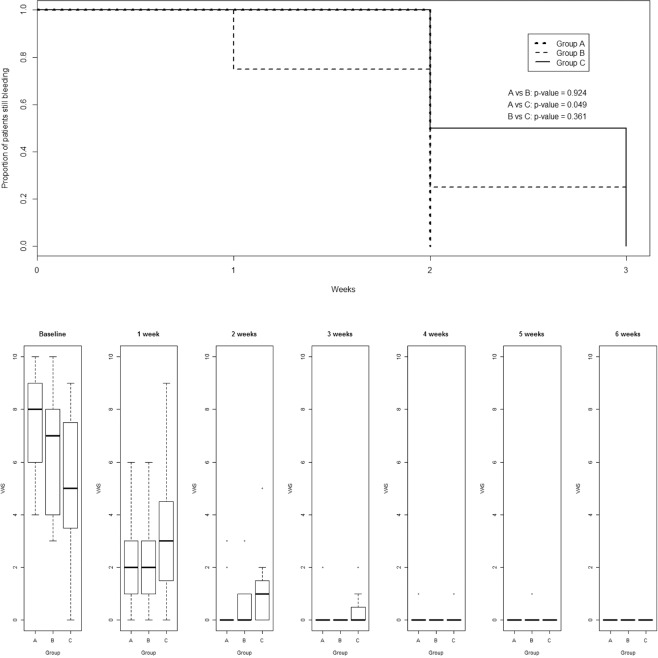
(**A**) (upper) The median time-to-stop bleeding of 34 patients with thrombosed hemorrhoids (TH), underwent incision and drainage. The median time was 1 week for Group (**A**), and 2 weeks for Groups (**B,C**), respectively. (**B**) (lower) VAS scores (pain evaluation) in 34 patients underwent incision and drainage for thrombosed hemorrhoids from baseline onward.

Phlebotonics showed no significant differences even if healing occurred in both Groups A and B within the second week. Group C’s patients healed at the end of the fourth week (Fig. 3A).

VAS scores of Groups A and B decreased significantly faster than Group C (A vs B, p < 0.001; A vs C, p < 0.001; B vs C, p < 0.001) (Fig. 3B and lower section of Table 2).

### Clinical effects of dietary regime

*Eighty-one (62.3%) of 130 patients with grade II-III-IV HD* were affected by *persistent constipation*. Dietary regime resolved constipation up third to fourth week. Four of them (3.1%) complained constipation at the end of the fourth week. None at the end of the fifth (upper section of Table 3).

**Table 3:** Constipation (yes/no) and BMI monitoring of investigated population.

grade II-III-IV HD (n = 130)	Patients	Group A	Group B	Group C
**Constipation**				
Baseline	81 (62.3%)	23 (51.1%)	27 (58.7%)	31 (79.5%)
2 weeks	63 (48.5%)	16 (35.6%)	24 (52.2%)	23 (59.0%)
4 weeks	4 (3.1%)	0 (0.0%)	1 (2.2%)	3 (7.7%)
6 weeks	0 (0.0%)	0 (0.0%)	0 (0.0%)	0 (0.0%)
		A vs B = ns A vs C = ns	B vs C = ns	
**BMI**				
Baseline	25.6 ± 4.6	25.7 ± 4.5	25.1 ± 4.3	26.1 ± 4.5
2 weeks	25.0 ± 4.2	25.1 ± 4.2	24.5 ± 4.0	25.6 ± 4.3
6 weeks	24.1 ± 4.9	24.2 ± 5.3	23.9 ± 3.8	24.3 ± 5.7
**Hemorrhoidectomy (n = 31)**				
**Constipation**				
Baseline	23 (74.2%)	7 (70.0%)	6 (66.7%)	10 (83.3%)
2 weeks	17 (54.8%)	4 (40.0%)	4 (44.4%)	9 (75.0%)
4 weeks	2 (6.5%)	1 (10.0%)	0 (0.0%)	1 (8.3%)
6 weeks	1 (3.2%)	0 (0.0%)	0 (0.0%)	1 (8.3%)
		A vs B = ns A vs C = ns	B vs C = ns	
**BMI**				
Baseline	24.8 ± 3.3	23.6 ± 3.2	25.6 ± 3.9	25.3 ± 3.0
2 weeks	24.4 ± 3.3	23.1 ± 3.0	25.2 ± 4.0	24.9 ± 2.9
6 weeks	23.9 ± 3.1	22.7 ± 2.6	24.6 ± 4.0	24.4 ± 2.7
**Hemorrhoidal Thrombosis (n = 34)**				
**Constipation**				
Baseline	19 (55.9%)	7 (41.2%)	7 (77.8%)	5 (62.5%)
2 weeks	18 (52.9%)	7 (41.2%)	6 (66.7%)	5 (62.5%)
4 weeks	2 (5.9%)	0 (0.0%)	1 (11.1%)	1 (12.5%)
6 weeks	0 (0.0%)	0 (0.0%)	0 (0.0%)	0 (0.0%)
		A vs B = ns A vs C = ns	B vs C = ns	
**BMI**				
Baseline	23.1 ± 3.8	23.4 ± 4.1	22.2 ± 3.7	23.7 ± 3.6
2 weeks	25.0 ± 4.2	25.1 ± 4.2	24.5 ± 4.0	25.6 ± 4.3
6 weeks	24.1 ± 4.9	24.2 ± 5.3	23.9 ± 3.8	24.3 ± 5.7

#### Patients who underwent hemorrhoidectomy (n = 31)

At baseline, 23 patients (74.2%) complained constipation. At the sixth week, 1 member of Group C (8.3%) showed *persistent constipation* (middle section of Table 3).

At the end of the fourth week of treatment, two (5.9%) of the *thirty four patients with HT* were still constipated. At the end of the sixth week, nobody was constipated (lower section of Table 3). Diet regime heals constipation within the fourth week^9–12,23,37,38^.

### Adverse reactions at phlebotonics

No adverse reactions were observed neither in patients treated with flavonoids^3,4,8,13–20^ nor in patients treated with Ca^21,22^. All patients completed the study with a good compliance. None was lost at the follow-up for side effects or nonmedical reasons. Phlebotonics were well tolerated. One patient complained of burning from Ca ointment that was suspended.

### Adverse reactions at medicated soap

No adverse reactions were observed. All patients showed a favorable compliance.

### Sensibility to the phlebotonics

**Figure 4:**
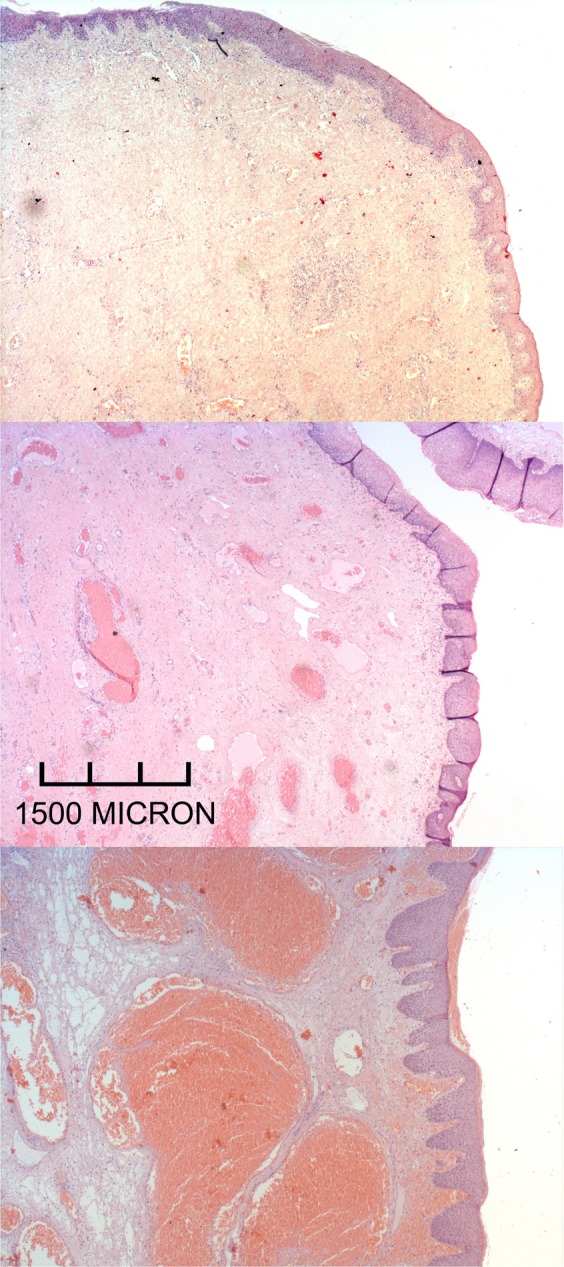
(**A**) (upper) Histology of a nodule characterized by diffused fibrosis and involutional aspects of the venous vessels. They appear rarefied, slightly ectatic, and congested (×2.5 magnifications). (**B**) (middle) Venous ectasia in the transition zone of the anorectal junction. It appears slightly fibrous. Venous vessels also appear slightly involuted in a patient treated with *Centella asiatica* (×2.5). (**C**) (lower) Hemorrhoidal nodule whose stromal axis is occupied by markedly ectatic venous vessels congested with extensive perivascular hemorrhages without fibrosis. Venous vessels appear slightly involuted (×2.5). The microphotographs and charts were created by contributing authors.

At the end of the second week checkup, *grade IV chronic bearings*’ volume was reduced. Moreover, the most relevant reduction was observed in Group A in comparison with Groups B and C. In fact, piles started to reduce with an understaging from IV to III grade Goligher’s classification (Fig. 1A,B). Patients who did not experienced an appreciable symptomatology improvement and understaging of piles underwent hemorrhoidectomy. Patients who were administered with flavonoids (with poor symptomatology improvement) showed anatomically diffuse fibrosis and involutional aspects of bearings venous vessels that appeared rarefied and slightly ectatic, with great reduction of vascular ectasia (Fig. 4A, ×2.5 magnifications). Figure 4B shows the venous ectasia of hemorrhoidal piles in the transition zone of the anorectal junction in patients who took Ca.

Figure 4C points out the specimen of a patient who underwent conservative treatment. This picture shows the stromal axis occupied by markedly ectatic and congested venous vessels and perivascular hemorrhages. Neither fibrosis nor involutional aspects of vessels were observed. As for sensibility to the therapy, it has been encoded as follows: 0/no sensibility; 1/sensibility; 2/doubtful sensibility. This code was used to build Table 4, that shows absolute frequencies and percentages related to the clinical efficacy of the therapies. With regard to III grade HD patients, a significant difference was observed in the percentage of sensitivity. The upper section of Table 4 reports on *III grade HD patients’* sensitivity and shows statistically significant differences among Groups of treatment (A vs C, p < 0.001; B vs C, p < 0.001; A vs B, p < 0.322). Twenty out of 22 patients of Group A and 11 out of 17 patients of Group B in comparison with each to other showed no significant differences, because they are both effective (Groups A vs B, p < 0.322). As for Group C, 2 of 14 patients showed a *clinical understaging* (A vs C, p < 0.001; B vs C p < 0.001). Finally, Table 4 shows the *association between fibrosis and flavonoids* (p = 0.008).

**Table 4:** Study of hemorrhoidal sensibility to the therapy and Histopathology results for grade IV bleeding hemorrhoidectomy underwent hemorrhoidectomy (n = 31).

Sensibility to the therapy 0-no 1-yes 2-not clear	Grade III hemorrhoidal disease			Grade IV bleeding hemorrhoids			Grade IV hemorrhoidectomy		
	0	1	2	0	1	2	0	1	2
A	2	20	0	5	10	3	5	3	1
B	4	11	2	11	10	3	7	1	1
C	11	2	1	13	5	0	9	2	0
*p-Value*	A vs C *p* < 0.001 B vs C *p* < 0.001 A vs B *p* = 0.322			*ns*			*ns*		
**Grade IV hemorrhoidectomy**									
**Groups**	**Fibrosis**			**Vascularization**			**Ectasia**		
	**1**	**2**	**3**	**1**	**2**	**3**	**1**	**2**	**3**
A	2 (16.7%)	8 (66.7%)	2 (16.7%)	3 (25.0%)	9 (75.0%)	0 (0.0%)	4 (33.3%)	6 (50.0%)	2 (16.7%)
B	4 (50%)	3 (37.5%)	1 (12.5%)	0 (0.0%)	5 (62.5%)	3 (37.5%)	2 (25.0%)	3 (37.5%)	3 (37.5%)
C	11 (91.7%)	1 (8.3%)	0 (0.0%)	2 (16.7%)	5 (41.7%)	5 (41.7%)	1 (8.3%)	4 (33.3%)	7 (58.3%)
*p-Value*	*p* = 0.008			*p* = 0.095			*p* = 0.290		

## Discussion

In this experience on 199 patients, of which *130 with HD bleeding at baseline*, the median time-to-stop bleeding was 2 weeks in Group A, 3 weeks in groups B and C with significant differences between the Groups (A vs B, p = 0.007; A vs C, p < 0.001; B vs C, p = 0.152) (Table 1, Fig. 1A). We observed better results in treated patients with flavonoids than in those treated with Ca and Controls. Current literature shows also that flavonoids mixture and PMFF have resulted to be effective in improving the proctoscopy appearance of hemorrhoids without significant differences between the two groups at 1 month (p = 0.87) and 6 months (p = 0.41)^15,36^.

As for *anal irritation*, after the first week, Groups A and B showed a similar improvement better than Group C.

Group A’s improvement became more relevant at the end of second week of treatment (Fig. 1B). The results are similar to La Torre outcomes for symptoms (*anal irritation* = pruritus and bleeding)^36^. Along medical checkups, grade IV and III HD resulted understated respectively to III and II grade. The upper section of Table 4 reports on *grade III HD patients’ sensitivity*. It shows statistical significant differences among Groups of treatment. In fact, twenty out of 22 patients of Group A, 11 out of 17 patients of Group B and 2 of 14 patients of Group C showed a *clinical understaging* (A vs C, p < 0.001; B vs C p < 0.001).

As for *31 cases with bleeding grade III-IV HD underwent hemorrhoidectomy*, the histomorphometry revealed that the type of administered therapy influences the volume of anal cushion, vessels’ number and ectatic vessels number for microscopic field with a strong association to flavonoids (*p* = 0.008)^8,13–22^ (lower section of Table 4). Moreover, enrolled patients weren’t exposed to other therapies.

This caution, together with the careful application of exclusion criteria, allows us to think that histomorphometry findings are related to phlebotonics effects.

*Cho*^20^ and other authors^21,22^ documented that “*aside from flavonoids, Ca stimulates collagen type-1 synthesis, the production and accumulation of new extracellular matrix (which improves vascular-connective tissue deposition) and repair processes that act on microbial and leucocyte enzymatic hydrolysis to shield collagen*^20–22^”.

Sitia *et al*. reported that “*autophagy is a process that sequestrates cellular waste materials such as damaged proteins and organelles by means of autophagosomes*^39^”. “*These are delivered to the lysosomes which degrade them into reusable monomers such as amino acids*^39,40^”.

Gomez-Sintes *et al*. reported that “*autophagic cell death has remodelled tissues, cardiac included*^41^”. Dunlop *et al*. stated that “*the kinase mammalian target of rapamycin (mTOR), located on the lysosomal membrane, is the key player in autophagy process*^42,43^”.

Li *et al*. reported that “*the activation of mTOR pathways, including phosphatidylinositol-3 kinase (PI3K)/Akt/mTOR pathway or adenosine monophosphate-activated protein kinase (AMPK)/mTOR pathway could inhibit the autophagic response*^40^”. In the “*restriction of growth factors, phosphorylation of the protein kinase Akt decreases giving rise to the induction of autophagy*^40^”.

Moreover, the results let suppose that resected patients probably started the conservative treatment too late to get understaging and to prevent the intervention.

Furthermore, Li *et al*. reported that “*hepatic fibrosis is a self-repairing process of liver injury, characterized by excessive accumulation of extracellular matrix (ECM)*^40^”. On one hand, Li-Shuang *et al*. stated that “*chronic hepatocyte injury, inflammatory response, secretion of inflammatory factors and accumulation of inflammatory leukocytes contribute to hepatic fibrosis*^44^”. On the other hand, they noticed that “*inflammatory environment can drive hepatic stellate cells (HSCs) from resting to activating state, which promotes their rapid expansion, massive synthesis and excessive of ECM*^44^”.

Due to the “*central position of HSCs in the development of hepatic fibrosis*”, numerous attempts proved that HSCs block or activation is a possible strategy for hepatic fibrosis treatment^44^. Notably, the inflammation creates a vicious cycle that makes worse the hepatic diseases^44^. In the initial phase of hepatic injury, “*cell death triggers inflammation, which contributes to resetting cellular debris*^44^”. This process promotes “*liver regeneration and restoring hepatic architecture and function*^43^”.

Li *et al*. reported that “*rutin has vitamin P-like and anti-inflammatory effect against hepatic fibrosis in TAA-induced mice and HSCs stimulation by means of TGF-β*^44^”. In this regard, Sharma *et al*. stated that “*rutin has also the functions of maintaining vascular resistance reducing permeability and brittleness*^45^”. Khan *et al*. discovered that “*rutin could inhibit peroxide formation by reducing the iron-mediated free radicals*^46^”. Acquaviva *et al*. proved that “*it improves the occurrence of certain injuries and inflammation by reducing vascular permeability*^47^”. Rutin can attenuate cholestasis related to liver injury. Reported studies results are compliant with the present paper. In fact, it was observed the volume reduction of anal cushions, vessels number for microscopic field reduction and number of ectatic vessels reduction with a strong association between histomorphometry and flavonoids administration (*p* = 0.008)^8,13–22^.

At the moment, both flavonoids and Ca are considered semi-essential nutrient supplements that can be purchased over-the-counter. For these reasons, such compounds should be classified as medications.

As for *34 patients with HT*, the comparison between treatments did not show significant differences, even if healing occurred within the second week in both Groups A and B. On the contrary, Group C healed at the end of the fourth week (Fig. 3A). VAS scores for pain in Groups A and B decreased significantly faster than Group C (A vs B, p < 0.001; A vs C, p < 0.001; B vs C, p < 0.001) (Fig. 3B, Table 2). The results match with Perera’s^14^ outcomes (Fig. 3A,B).

An English research has shown that 51% of patients with hemorrhoids are also affected by constipation^9^.

This study shows that *constipation in grades II-III-IV HD* patients was observed in 62.3% (Table 3). A *diet regime* rich in water and boiled-vegetable-fiber is a strong recommendation with moderate-quality evidence^3^. This diet regime heals constipation within the fourth week (Group A) and a long term investigation should be addressed by this item. *Medicated soap* (with Ca among other herbal compounds) couldn’t have a confounding effect on study’s results for the little dose and also because the treatment was administered to all enrolled patients. On the contrary, Group B (which received Ca extracts both orally and topically) would have had a better result than Group A both in bleeding, pain and histomorphometry.

Brusciano *et Al*. reported that 1470-nm diode laser (Biolitec® Jena, Germany) hemorrhoidoplasty (LHP), a minimal invasive procedure for II–III degrees HD treatment, caused the shrinkage of hemorrhoidal piles^48,49^. This is an interesting opportunity that proctologists have to consider for the treatment of lower HD grades as well as the conservative treatment. Surprisingly, it has been observed that conservative treatment determines the shrinkage of higher bearings grades in comparison to the laser procedure. The only unfavorable aspect is that the conservative procedure requires more compliance by patients and their education. In II-III-IV grades HD, it wasn’t noticed postoperative pain, no anal wounds, no surgical time and a discomfort. Moreover, 100% of patients treated with conservative protocol continued their daily activities along with the treatment contrary to the laser procedure that required two days off.

A long term study on administration of phlebotonics could be useful to verify beneficial effects persistence likewise side effects, if any, not observed in this short time experience.

Sometimes, young women suffering II-III grade HD complaint rectum bulges into the back wall of the vagina, also called rectocele, one type of pelvic organ prolapse. It happens because of supporting ligaments and muscles weaken in the pelvic floor and/or incoordination of pelvic muscles. In these cases, it is important to choose a conservative treatment and ask for an anorectal manometry to measure pressures and sensations of the patient. It is well known that biofeedback treatment allows, through a probe, a device and a dedicated monitor, the visualization and representation of the contraction and relaxation activity of the anal sphincter. Before the beginning of any kind of biofeedback or rehabilitative treatment, the physicians should assess and verify the patient’s proprioceptive consciousness of the anal sphincter. In coloproctological evaluation with Clevaland Clinic Fecal Incontinence score, Murad-Regadas *et al*. considered the anamnesis of previous anal surgery, hysterectomy and previous vaginal deliveries predictive factors that influence the effectiveness of the rehabilitative treatment^50^.

Probably, in presence of muscular synergies without selective contraction of anal sphincter, a high percentage of the failed patients would have benefited of a previous electrostimulation treatment. This choice would have helped to acquire patients’ consciousness of the anal area^51^. Rehabilitation treatment should start with a patient’s re-educational phase in order to clearly explain and clarify that chest, abdomen, vertebral column and perineum act as different parts of a same whole (an imaginary cuboid), especially in people with defecation disorders that causes HD^49^. Therefore, the above-mentioned abnormal contraction of abdominal muscles is the demonstration that distant areas from pelvic floor contribute to its function. This clinical-physiatric approach aims to improve an altered bodily function and prepare the patient toward an active role during the healing process^52^. Coupled with a dietary regime, this procedure can heal constipation and anal problems that frequently don’t require surgical treatments.

### Limitations

A greater number of consecutive patients were enrolled including all the patients treated until the end of the follow-up period (n = 130 subjects with *subacute and chronic symptomatology grades II-III-IV HD* randomized in three arms).

Moreover, in the present paper, there were also reported results of patients diagnosed with:

- *bleeding grade IV hemorrhoids underwent hemorrhoidectomy* (n = 31 randomized in three arms);
- *bleeding grade II hemorrhoid underwent rubber band ligation* (n = 4 randomized in three arms but not analyzed because of insufficient number of patients);
- *hemorrhoidal thrombosis underwent incision and drainage procedure* (n = 34 randomized in three arms).

All these three diagnoses weren’t included in the initial protocol design. In any case, the enrolled patients with these three adjunctive diagnoses were randomized in three arms and followed the same procedure and treatments as designed for the protocol (designed for diagnosis of subacute and chronic symptomatology grades II-III-IV HD).

As for n = 130 subjects with *subacute and chronic grades II-III-IV HD* randomized in three arms, the sample size N calculated should have been 105 patients of which 35 patients in the control Group C, and 70 in the experimental group (35 patients in Group B and 35 in Group A). The enrolled patients were more: Group A n = 45 treated with flavonoids, Group B n = 46 treated with Centella and Group C n = 39 (upper section of Table 1).

As for *bleeding grade IV hemorrhoids underwent hemorrhoidectomy n* = ***31*** and *hemorrhoidal thrombosis underwent incision and drainage procedure n* = *34*, the cases enrolled in the study period were insufficient, but results clearly showed the same trend of subacute and chronic grades II-III-IV HD. This is an interesting result to share with the scientific community.

Moreover, this research results are true in the short time. A longer time control could be interesting to prove if correct feeding habits are preserved along the time. The enrolled patients were advised to avoid hot and red peppers despite Altomare’s statement^53^. It would be useful to clarify this item definitively.

## Conclusion

These results and the analysis of literature^8,14–16^ agree that flavonoids are effective treatment in terms of shorter time for disappearance of bleeding and anal irritation in patients with grades II-III-IV HD. Bleeding heals by the first week if patients are treated with flavonoids, whereas either flavonoids or Ca^21^ were equally effective on irritation. Both have better performance than traditional treatment. Patients, who underwent hemorrhoidectomy, and those incised and drained for HT probably started treatments too late to avoid intervention. There were neither complications nor toxicity in the enrolled patients and phlebotonics studied resulted safe and tolerable. As for efficacy, grade III-IV HD patients, who took either flavonoids or Ca, showed fibrosis and understaging aspects of their hypertrophic piles^8,14–16^. Finally, at the moment, both flavonoids and Ca are considered semi-essential nutrients and are purchased over-the-counter. The use of these products in clinical practice should be reconsidered and such compounds should be classified as medication. A diet regime rich both in water and boiled-vegetable-fiber is needed to resolve constipation in 4 weeks^9–12,23,37,54^.

## Materials and Methods

One hundred ninety nine patients, both males and females, were enrolled for this study. All of them followed a diet regime and hygienic care protocol over the full period of 42 days and data were collected. *One hundred thirty Golligher’s classification grade II-III-IV HD* both “Treated” underwent treatment with phlebotonics [randomized in Group A (n = 73, flavonoids), and B (n = 66, Ca)] and “Controls” randomized in Group C (n = 60) received the routine treatment without phlebotonics.

*Thirty-one patients with bleeding grade IV* did not heal within 6 weeks with conservative treatment. They underwent a standard Ferguson-type hemorrhoidectomy (under epidural anesthetic), that involves ligature of the three main pedicles by transfixing the tip with poliglactin suture and mucosa reconstruction.

*Thirty-eight patients with acute hemorrhoidal thrombosis (HT)* underwent local anesthesia, incision and drainage (Table 1).

### Patients randomized in Group A

Treated patient randomized in Group A (n = 73) (chronic HD, hemorrhoidectomy and HT) received additional oral flavonoids tablets 300 mg (Flavonil® containing Rutin, borago officinalis) twice a day for 15 days and topical administration of 3 g of ointment inside the anus (Flavonil® ointment containing bromelain, borago officinalis, aesculus hippocastanum, Hamamelis virginiana, ruscogenine) once a day after hygienic care procedure.

### Patients randomized in Group B

Likewise, Treated patient randomized in Group B (n = 66) received additional oral *Ca* tablets 60 mg (Centella Complex® containing triterpenic acids and asiaticoside) twice a day for 15 days and 3 g of Proctocella® ointment (containing Centella Asiatica, Arnica, Aloe) (Table 1)^21,22^.

### Patients randomized in Group C

Control patients (Group C, n = 60) received routine treatment without phlebotonics support.

Diet regime was spread over 5 meals each day to all patients, low in salt, rich in water and boiled vegetables^9–12^. No seasonings, spice, alcohol, chocolate, balanced as Recommended Daily Allowance^37^.

### Hygienic cares

It consist in avoiding toilet paper use, washing the anal region with lukewarm water and medicated soap (Rectalgan Mousse® containing Ca, Hamamelis virginiana, propolis, Salvia officinalis, Aloe barbadensis, and sodium hyaluronate), drying the anal area with a soft towel. Any contemporary topic therapy was not accepted.

### The inclusion criteria

Both males and females, aged between 18 and 85, complained with hypertrophic piles both chronic and acute HD, anal irritation (pruritus^36^ for not operated patients), pain (for operated patients), bleeding during defecation. Patients were enrolled only with at least two checkups.

### The exclusion criteria

The criteria were: allergy to flavonoids or Ca; inflammatory bowel disease (Crohn disease, Ulcerative Colitis); rectal and anal cancer; tuberculosis; presumed or confirmed pregnancy or lactation; alcohol/drugs dependence, as well as patients with mental disorders; cardiovascular and/or metabolic problems (decompensated diabetes and hypercholesterolemia); those with thrombocytopenia or were taking oral anticoagulant therapy; previous coloproctological surgery and associated complications; those who were taking antibiotics or antiviral, thyroid hormones, anticonvulsants, antidepressants, anticholinergics, antihistamines, statins, diuretics, steroidal anti-inflammatory drugs, nonsteroidal anti-inflammatory drugs, antipyretics.

### Bleeding, anal irritation, and pain assessment

The intensity of anal irritation (pruritus in patient who underwent conservative treatment) or pain (for operated patients) were measured by the visual analogue scale (VAS). The active bleeding (yes/no) was checked at baseline (day 0) and also during the following checkups (from day 0 up to day 42).

### Study design

The study is a comparative randomized clinical trial with each other of clinical effects of flavonoids and Centella asiatica and also in comparison to the control group (conservative treatment) in patients with HD or those underwent surgery (i.e. post-hemorrhoidectomy, and post-HT incision and drainage).

The study design was approved both by the Council of *Department of General Surgery and Specialties “Paride Stefanini”, Sapienza University of Rome, Rome, 00161, Italy (June, 4, 2013)*, and by Ethics review board of University of *Sapienza University of Rome, Azienda Ospedaliero-Universitaria Policlinico Umberto I, Viale del Policlinico, 155, 00161, Rome, (*+*390649979822, e-mail: comitato.etico@policlinicoumberto1.it), Rome, Italy* (Prot n 174/18; 02/27/2018).

Then, the protocol of this research was published online (www.clinicaltrials.gov, identifier number NCT03569930, the full date of registration is 26/06/2018) and carried out according to principles of Helsinki Declaration and Good Clinical Practice from June 4, 2013, up to December 30, 2018.

Patients were informed on study’s strategies and they signed the informed consent before enrollment started. Healing was defined as no bleeding and no *anal irritation* in patients who underwent conservative treatment. In operated patients, healing was defined as bleeding and *pain* disappearance. Patients enrolled were monitored with an observation period of 42 days. This randomized clinical trial was performed on outpatients with HD that presented voluntarily at the Proctology Clinic of the *Department of General Surgery and Specialties “Paride Stefanini”*. The first visit consisted in the enrollment and patients were warned to expect some phone calls to be interviewed. In operated patients, the first postoperative visit was performed at day seventh (D7) after surgery and hospital discharge. Two phone calls (at the end of the second and third week made by a trained male nurse: Mr. Sebastiano Crisci) and two visits (one on the seventh and the second one on the twenty eighth day) were performed.

During the first visit, the perianal region was inspected to assess the condition of surgical wounds. Digital rectal examination (and anoscopy if needed) was performed at D28. Patients received daily diary cards on which they were asked to report the worst irritation sensation experienced each day with a VAS scale of 1 to 10. Patients (surgical and conservative treatments) were asked to report about bleeding, understaging (hemorrhoidal cushions volume reduction in conservative treatment) and about the hardness of the fecal mass, regularity of defecation, and healing of chronic constipation. Furthermore, they were asked about side effects (headache, blood pressure changes, allergic reactions), if any.

### End points

*The primary end* point was the time-to-stop bleeding estimated by Kaplan-Meier method. *The second outcome* measure was the disappearance of anal irritation (not operated patients) and pain (operated patients). The efficacy of the therapy administered was evaluated by assigning an encoding (0/no sensibility; 1/sensibility; 2/doubtful sensibility) attributed to findings observed at the medical check-ups.

### Histological analysis

Blind sampling and histomorphometry of histological specimens of operated patients with bleeding grade IV HD were studied. Investigated parameters were fibrosis (involution of rectal cushions into fibrotic tissue), venous vascularization (vessels number for microscopic field) and vessel ectasia (number of ectatic vessels). Sample specimens of all groups were fixed in 10% formaldehyde and processed by standard method. All tissues were embedded in paraffin blocks. Sections were stained with hematoxylin and eosin (H&E). Mentioned parameters were evaluated by examination under a light microscope and classified on a numerical index, 1 to 3.

### Efficacy of diet

Efficacy of diet regime rich in water and boiled vegetables was evaluated in terms of constipation healing and BMI assessed by measuring body weight with own home available instrument in appropriate conditions^9–12,37^.

### Assessment of constipation

Wexner’s constipation scoring system was used to simplify constipated patients evaluation and management. A score (15 up to 30) greater than 15 established the constipation^9–12,23,37,54^.

### Safety of therapy, adverse events, reactions and side effects

Adverse events, reactions and side effects were investigated (see the published protocol) in all patients. Authors ensure that this Methods section includes availability of database with adequate experimental data to reproduce this work. Moreover, this method could be useful to prevent surgical treatment of hemorrhoids^27,36,39,55^.

### Statistics

In order to size the study and then choose an adequate sample size N, the formula proposed in Schoenfeld D. was used^38^. The enrolled number of patients was greater than those expected including all the treated patients until the end of the follow-up period (130 subjects with subacute and chronic symptomatology grades II-III-IV HD). Moreover, in order to enlarge the applicability of the protocol to other proctological diagnoses, the study considered the patients with bleeding grade IV hemorrhoids underwent hemorrhoidectomy, bleeding grade II hemorrhoid patients underwent rubber band ligation and hemorrhoidal Thrombosis underwent incision and drainage procedure who weren’t included in the study protocol.

A computer-generated randomization list was used to allocate patients to the three trial arms. Numerical data were summarized as mean and standard deviation or median and ranges, as appropriate. Categorical data were expressed as absolute and relative frequencies. The probability of achieving healing (time-to-stop bleeding within the 6 weeks of the study) was estimated by Kaplan-Meier method and Log-rank R tests with post-hoc analysis by Bonferroni method were used to compare Kaplan–Meier curves. The proportional hazards assumption was verified using scaled Schoenfeld residuals. The disappearance of *anal irritation* (not operated patients) and *pain* (operated patients) was studied by Kruskal-Wallis to evaluate changes in the VAS scores through multiple comparisons between paired subgroups assessed with the Nemenyi test. As for the efficacy, to compare the percentages of the non-sensitive/sensitive/doubtful subjects, in the various Groups of therapy and for the different diagnoses, was used the chi-square test with Bonferroni adjustment for multiplicity. As for the efficacy of therapy administered, it was evaluated by assigning an encoding (0/no sensibility; 1/sensibility; 2/doubtful sensibility) attributed to findings observed at the medical check-ups. Statistical significance was set at 0.05. All analyses were performed by the R software, version 3.5.0 (The R Project for Statistical Computing, Vienna, Austria).

## Supplementary information

Dataset 1, Dataset 2, Dataset 3, Dataset 4.

## Data Availability

database, anonymized data and associated protocols are promptly available to readers without undue qualifications in material transfer agreements.
